# Frailty, Surgical Time, and Surgical Complications Increase Length of Stay Following Large Vestibular Schwannoma Resection

**DOI:** 10.1002/ohn.70063

**Published:** 2025-10-31

**Authors:** Jason L. Steele, Nicole J. Ewer, Heather J. Smith, Samira Takkoush, Melissa Shuhui Lee, Richard H. Wiggins, Mana Espahbodi, Neil S. Patel, Richard K. Gurgel

**Affiliations:** ^1^ Rocky Vista University College of Osteopathic Medicine Ivins Utah USA; ^2^ University of Utah School of Medicine Salt Lake City Utah USA; ^3^ Department of Otolaryngology–Head and Neck Surgery University of Utah Salt Lake City Utah USA; ^4^ Department of Radiology and Imaging Services University of Utah Salt Lake City Utah USA; ^5^ Department of Neuroradiology Singapore General Hospital Singapore; ^6^ Department of Neuroradiology National Neuroscience Institute Singapore

**Keywords:** frailty, length of stay, nonhome discharge, vestibula schwannoma

## Abstract

**Objective:**

Patients with large vestibular schwannomas (VS) are at higher risk for adverse outcomes following microsurgical resection. *This* study aims to identify clinical and radiographic factors that may be predictive of adverse outcomes.

**Study Design:**

Retrospective chart review.

**Setting:**

Academic tertiary care center.

**Methods:**

A chart review of patients with Koos III or IV VS from 2016 to 2020 was conducted. Demographics, preoperative 11‐item modified frailty index (mFI‐11), and surgical information were collected: preoperative Koos score and the total tumor volume (TV) on T2 MRI sequences. Preoperative and postoperative tumor characteristics, symptoms, and House‐Brackmann (HB) facial nerve function were assessed. Outcomes included increased length of stay (LOS; defined as >4 days), nonhome discharge (NHD), and surgical complications. Statistical analysis was performed with SPSS version 29.

**Results:**

In total, 79 patients were identified; 54% were female and 86% were white. On univariable binary logistic regression analysis, factors associated with increased LOS included surgical time, TV, frailty (measured by mFI‐11), and any surgical complication. On multivariable analysis, only increased frailty (odds ratio [OR]: 12.99; 95% CI: 2.06‐81.91), surgical time (OR: 1.01; 95% CI: 1.00‐1.02), and having a surgical complication (OR: 5.95; 95% CI: 1.48‐23.87) were independent predictors of LOS. The only independent predictor of NHD was VP shunt placement after surgery (OR: 9.71; 95% CI: 1.18‐80.02). There were no other independent predictors of LOS, NHD, or surgical complications.

**Conclusion:**

Frailty, as measured by increased mFI‐11, surgical time, and surgical complications were independent predictors of increased LOS. VP shunt placement after surgery was an independent predictor of NHD.

Vestibular schwannomas (VS) are benign tumors derived from the Schwann cells of the eighth cranial nerve. They represent approximately 8% of all intracranial tumors and 85% to 90% of all cerebellopontine angle tumors (CPAs).^1^, ^2^, ^3^ The most common presentation is asymmetric or unilateral sensorineural hearing loss, with the VS diagnosed upon subsequent imaging. After imaging, tumors are graded using the Koos grading scale, ranging from 1 (intracanalicular only) to 4 (displacing brainstem, >3 cm).^4^ In larger tumors, mass effect can cause other symptoms, including trigeminal nerve dysfunction, cerebellar dysfunction, and hydrocephalus.^2^, ^5^

To relieve brainstem compression, the preferred treatment for large VS (greater than 3 cm) is microsurgical resection generally via a translabyrinthine or retrosigmoid approach.^2^, ^3^, ^6^ Depending on tumor size, location, and surgical approach, significant perioperative morbidity can occur.^7^ As a result, new screening tools and treatments are being developed in an effort to accurately predict and prevent postoperative morbidity and mortality while maintaining or improving treatment outcomes.^8^, ^9^, ^10^, ^11^ Adverse outcomes that have been investigated previously include cerebrospinal fluid (CSF) leak, cerebrovascular accident, facial nerve paresis or paralysis, hemorrhage, increased length of stay (LOS), and nonhome discharge (NHD), among others.

The purpose of this study is to evaluate factors predictive of adverse outcomes, specifically increased length of hospital stay, NHD, and surgical complications. Because patients are typically ambulatory at the time of discharge,^2^ both LOS and NHD were used as broad categories to encompass postoperative status and as an estimation of morbidity. Improving the ability to recognize factors associated with adverse outcomes will aid surgeons and patients in making sound decisions regarding treatment and expectations.

## Methods

After obtaining approval from the University of Utah Institutional Review Board (IRB_00045048), a retrospective chart review was conducted at a single tertiary academic care center. Inclusion criteria were subjects with a VS rated as Koos III or Koos IV on preoperative imaging who subsequently underwent microsurgical resection between 2016 and 2020. A Koos III tumor is defined as occupying the CPA and contacting the brainstem without brainstem compression. A Koos IV tumor is defined as tumors causing brainstem compression or displacement of nearby cranial nerves.^12^ Demographic information, including sex, race, ethnicity, age, and body mass index (BMI), was collected. Additional information gathered included total tumor volume (TV), postoperative House‐Brackmann (HB) scores for facial weakness, surgical approach, surgical time, Charlson Comorbidity Index (CCI) score, frailty scores as measured by the 11‐item modified frailty index (mFI‐11), LOS, discharge location, and surgical complications (CSF leak, stroke, hemorrhage, meningitis, and “other”). The mFI‐11 includes functionally dependent status, diabetes mellitus, chronic obstructive pulmonary disease or current pneumonia, congestive heart failure, myocardial infarction within the last 6 months, angina within 1 month or previous cardiac surgery/percutaneous coronary intervention, hypertension requiring medication, impaired sensorium, transient ischemic attack, cerebrovascular accident with deficits, and peripheral vascular disease. The CCI includes age, myocardial infarction, congestion heart failure, peripheral vascular disease, cerebrovascular accident or transient ischemic attack, dementia, chronic pulmonary disease, connective tissue disease, peptic ulcer disease, liver disease, diabetes mellitus, hemiplegia, moderate to severe chronic kidney disease, solid tumor, leukemia, lymphoma, and acquired immunodeficiency syndrome.

TV measurements were measured and calculated using Horos software (version 3.0; Nimble Co LLC), an open‐source 64‐bit medical image viewer based on OsiriX platform under the GNU Lesser General Public License. Heavily weighted T2 MRI sequences were used for measurements. Slice thickness had a median of 1.00 mm with an interquartile range of 0.67 to 3.00 mm. MRI images were imported into Horos from the PACS IntelliSpace Radiology 4.7 medical imaging interface and reviewed by a senior neuroradiologist (R.H.W.), a visiting radiologist (M.S.L.), a physician (S.T.), and two medical students (J.L.S., H.J.S.). The physician and medical students received focused training from the senior neuroradiologist on measuring axial cross‐sectional images. Pearson Correlation coefficients were run to assess interrater reliability (N = 20), which showed good agreement for TV (*r* = 0.991, *P* ≤ .001) and Koos score (*r* = 0.931, *P* ≤ .001). Three‐dimensional representations of each tumor were generated by Horos from the aggregated images.

Statistical analyses were conducted using IBM Statistical Package for Social Sciences (SPSS) version 29 (International Business Machines [IBM Corp.]). Univariable binary logistic regressions were performed for the following adverse outcomes (dependent variables): increased length of stay (>4 days), NHD, and surgical complications. Multiple definitions of increased length of stay have been used in the literature, with no current unified framework.^13^, ^14^, ^15^, ^16^ The definition of >4 days was used because this most closely aligned with the mean and median of our data, similar to Long et al.^14^ The following complications or postoperative symptoms (independent variables): age at surgery, sex, BMI, frailty, TV, surgical approach, surgical time, having any surgical complication, ventriculoperitoneal (VP) shunt placement after surgery, postoperative facial nerve function, and postoperative dizziness. Increased length of stay was defined as greater than 4 days. Facial nerve function was divided into two groups based on HB scores (HB 1‐2, HB 3‐6). Surgical complications included stroke, hemorrhage, CSF leak, meningitis, and “other” category for any issue deemed to be a complication. Multivariable binary logistic regression was then performed for all variables significant on univariable analysis, with near‐significant variables being used as needed. The Hosmer‐Lemeshow Test for goodness of fit was used for multivariable analysis, with a *P*‐value of <.05 indicating poor fit. The results are reported as odds ratios (ORs) with corresponding 95% confidence intervals (95% CIs). *P*‐values < .05 were considered significant.

## Results

### Demographics

After review, 79 patients met the criteria and were included for statistical analysis. See Table 1 for demographics and other characteristics.

**Table 1:** Patient Demographics and Characteristics

Patient characteristics	Results
Mean age at surgery	48.68 y
Sex	54.4% female
Race	86.1% white
Average BMI	29.67
Frail (mFI‐11 ≥ 2)	12.7%
Mean total tumor volume on T2	7.54 cm^3^
Surgical approach	
Translabyrinthine	77.2%
Retrosigmoid	22.8%
Type of resection	
Gross total	88.6%
Near total	8.9%
Subtotal	2.5%
Mean surgical time	329.3 min
VP shunt placement after surgery	5.1%
Postoperative House‐Brackmann > 2	40.5%
Postoperative dizziness	58.1%
Home discharge	88.6%
Increased length of stay (>4 d)	30.4%
Any surgical complication	22.8%

Abbreviations: BMI, body mass index; mFI‐11, 11‐item modified frailty index; VP, ventriculoperitoneal.

### Increased Length of Stay

**Figure 1:**
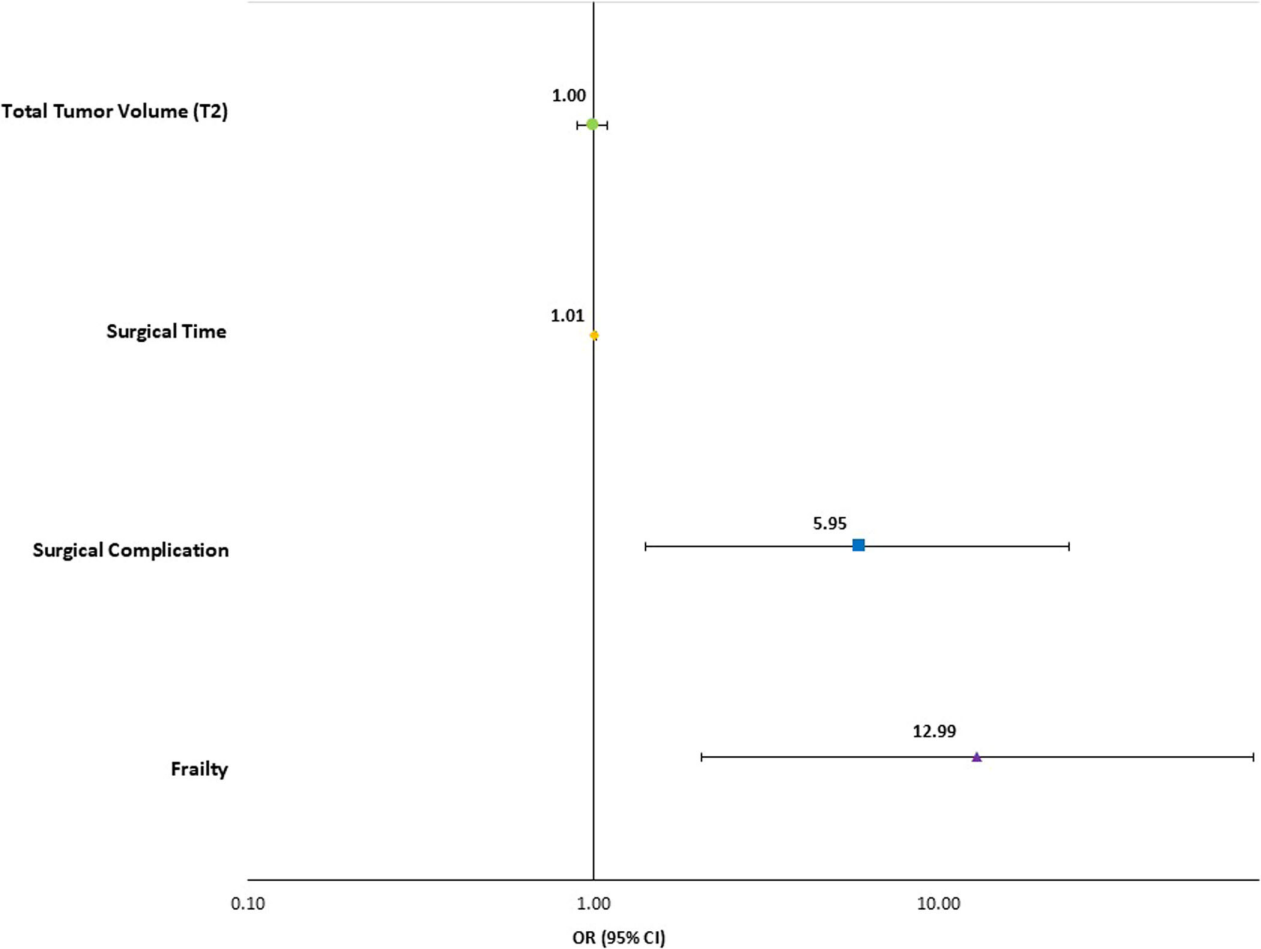
Odds ratios with 95% confidence error bars for multivariable analysis for length of stay. OR, odds ratio.

On univariable analysis, five characteristics were significantly associated with an increased LOS. These were frailty (OR: 7.14; 95% CI: 1.66‐30.71), TV (OR: 1.09; 95% CI: 1.02‐1.17), surgical time (OR: 1.01; 95% CI: 1.00‐1.02), and any surgical complication (OR: 8.17; 95% CI: 2.55‐26.20). Table 2 shows all odds ratios with 95% CI on univariable analysis. On multivariable analysis including the factors significant on univariable analysis (frailty, TV, surgical time, and any surgical complication), only frailty (OR: 12.99; 95% CI: 2.06‐81.91), surgical time (OR: 1.01; 95% CI: 1.00‐1.02), and any surgical complication (OR: 5.95; 95% CI: 1.48‐23.87) remained statistically significant. Table 3 shows all multivariable logistic regression analysis results for increased LOS. Hosmer‐Lemeshow Test for goodness of fit was 0.137. See Figure 1 for a graphical representation of LOS results.

**Table 2:** Univariable Logistical Regression Regarding Independent Factors Related to Increased Length of Stay (Greater Than 4 Days)

	Increased length of stay OR (95% CI)	*P*‐value
Age at surgery	1.00 (0.97‐1.04)	.906
Sex	.98 (.38‐2.58)	.975
Race	1.19 (0.66‐2.11)	.566
BMI	1.03 (0.96‐1.11)	.465
Frailty	7.14 (1.66‐30.71)	.008*
Charlson Comorbidity Index	2.42 (0.76‐7.69)	.134
Total tumor volume on T2	1.09 (1.02‐1.17)	.016*
Surgical approach	1.19 (0.39‐3.68)	.757
Surgical time	1.01 (1.00‐1.02)	.002*
Surgical complication	8.17 (2.55‐26.20)	<.001*
VP shunt placement after surgery	7.71 (0.76‐78.39)	.084
Postoperative House‐Brackmann	1.75 (0.66‐4.62)	.258
Postoperative dizziness	3.50 (0.99‐12.32)	.051

Abbreviations: BMI, body mass index; OR, odds ratio; VP, ventriculoperitoneal.

* Denotes statistical significance.

**Table 3:** Multivariable Logistical Regression Analysis Regarding Independent Factors Related to Increased Length of Stay

	Increased length of stay OR (95% CI)	*P*‐value
Frailty	12.99 (2.06‐81.91)	.006*
Surgical complication	5.95 (1.42‐23.87)	.009*
Surgical time	1.01 (1.00‐1.02)	.020*
Total tumor volume on T2	1.00 (0.90‐1.10)	.277

Abbreviation: OR, odds ratio.

* Denotes statistical significance.

### Nonhome Discharge

On univariable analysis, one characteristic was significantly associated with an NHD: VP shunt placement after surgery (OR: 9.71; 95% CI: 1.18‐80.02). No other factors were statistically significant on univariable analysis. Table 4 shows all univariable logistic regression analysis results for NHD. Table 5 shows multivariable logistic regression analysis results for NHD, which included the variable *Frailty, which had near significance on univariable analysis* (*P* = .064). Hosmer‐Lemeshow Test for goodness of fit was 0.823.

**Table 4:** Univariable Logistical Regression Regarding Independent Factors Related to Nonhome Discharge

	Nonhome discharge OR (95% CI)	*P*‐value
Age	1.02 (0.97‐1.07)	.459
Sex	1.78 (0.41‐7.71)	.438
Race	1.12 (0.60‐2.11)	.716
BMI	1.02 (0.92‐1.129)	.719
Frailty	4.50 (0.922‐22.08)	.064
Charlson Comorbidity Index	2.42 (0.53‐11.04)	.255
Total tumor volume on T2	1.02 (0.95‐1.11)	.542
Surgical approach	0.39 (0.05‐3.34)	.390
Surgical time	1.00 (0.99‐1.01)	.281
Surgical complication	3.20 (0.76‐13.50)	.113
VP shunt placement after surgery	9.71 (1.18‐80.02)	.035*
Postoperative House‐Brackmann	0.71 (0.16‐3.06)	.643
Postoperative dizziness	2.40 (0.44‐12.98)	.309

Abbreviations: BMI, body mass index; OR, odds ratio; VP, ventriculoperitoneal.

* Denotes statistical significance.

**Table 5:** Multivariable Logistical Regression Analysis Regarding Independent Factors Related to Nonhome Discharge

	Nonhome discharge OR (95% CI)	*P*‐value
Frailty	4.29 (0.80‐22.91)	.089
VP shunt placement after surgery	9.17 (1.01‐82.82)	.049*

Abbreviations: OR, odds ratio; VP, ventriculoperitoneal.

* Denotes statistical significance.

### Surgical Complications

**Figure 2:**
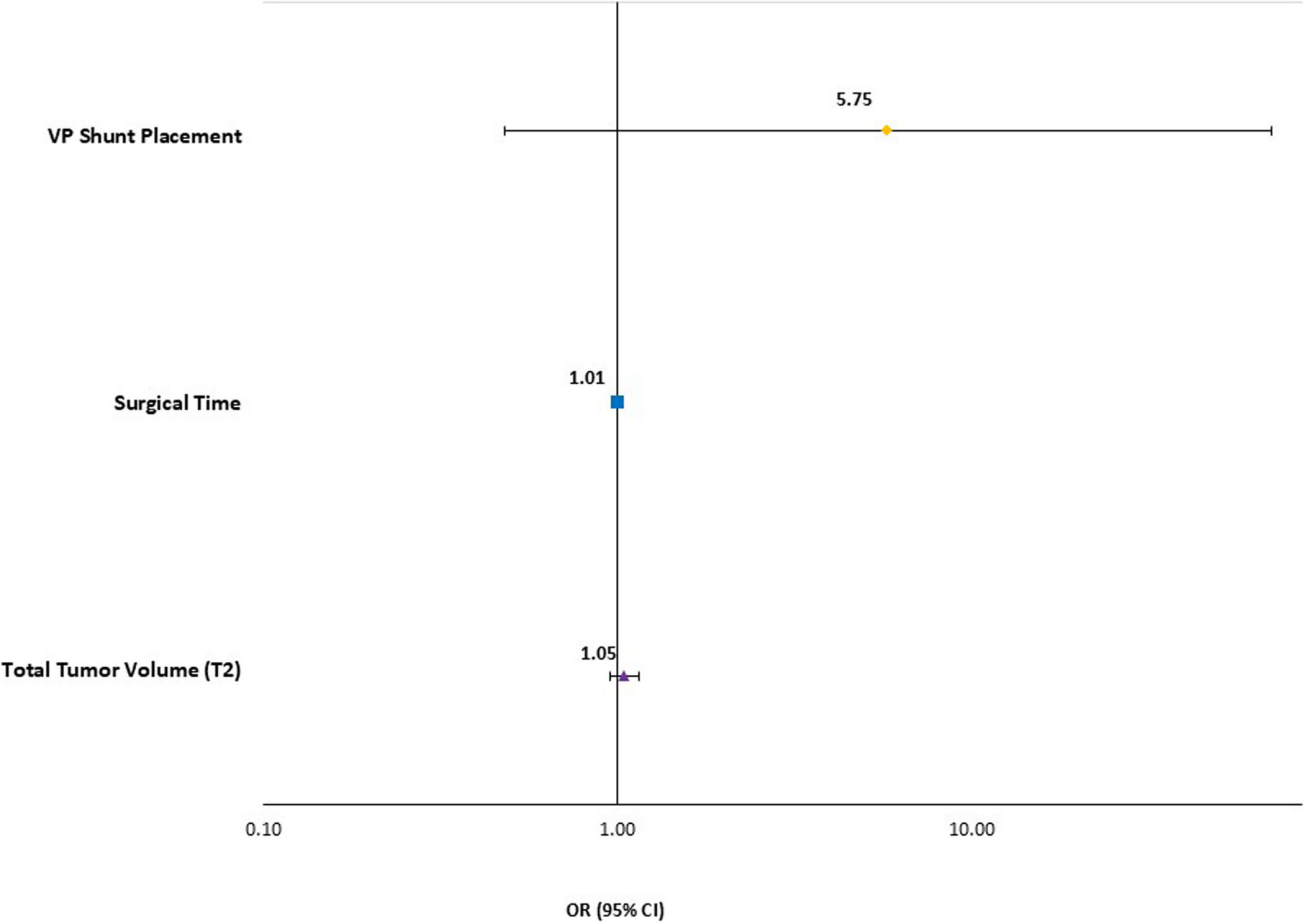
Odds ratios with 95% confidence error bars for multivariable analysis for surgical complications. OR, odds ratio; VP, ventriculoperitoneal.

On univariable analysis, three factors were significantly associated with having any surgical complication: TV (OR: 1.10; 95% CI: 1.02‐1.18), surgical time (OR: 1.01; 95% CI: 1.00‐1.02), and VP shunt (OR: 12.00; 95% CI: 1.16‐123.68). No other factors were statistically significant on univariable analysis. Table 6 shows all univariable logistic regression analysis results for having any surgical complication. Of the three, none remained statistically significant on multivariable analysis; none of these factors remained statistically significant (Table 7). Hosmer‐Lemeshow Test for goodness of fit was 0.586. See Figure 2 for a graphical representation of surgical complication results.

**Table 6:** Univariable Logistical Regression Analysis Regarding Independent Factors Related to Having a Surgical Complication

	Surgical complications OR (95% CI)	*P*‐value
Age	0.97 (0.93‐1.01)	.11
Sex	0.59 (0.21‐1.71)	.336
Race	1.15 (0.70‐1.89)	.583
BMI	0.96 (0.88‐1.05)	.373
Frailty	2.62 (0.65‐10.56)	.176
Charlson Comorbidity Index	0.82 (0.20‐3.28)	.775
Total tumor volume on T2	1.10 (1.02‐1.18)	.013*
Surgical approach	2.04 (0.64‐6.55)	.230
Surgical time	1.01 (1.00‐1.02)	.017*
VP shunt placement after surgery	12.00 (1.16‐123.68)	.037*
Postoperative House‐Brackmann	‐	‐
Postoperative dizziness	‐	‐

Abbreviations: BMI, body mass index; OR, odds ratio; VP, ventriculoperitoneal.

* Denotes statistical significance.

**Table 7:** Multivariable Logistical Regression Analysis Regarding Independent Factors Related to Surgical Complications

	Surgical complications OR (95% CI)	*P*‐value
Total tumor volume on T2	1.05 (0.95‐1.15)	.361
Surgical time	1.01 (1.00‐1.01)	.193
VP shunt placement after surgery	5.75 (0.48‐69.70)	.169

Abbreviations: OR, odds ratio; VP, ventriculoperitoneal.

## Discussion

This single‐institution study evaluated factors predictive of adverse outcomes from microsurgical resection of large VS, specifically increased LOS, NHD, and surgical complications. Three factors showed a significant association with increased LOS (frailty, surgical time, and any surgical complication). VP shunt placement was associated with NHD. No factors were associated with surgical complications after multivariable regression. These findings add to the growing body of literature supporting the use of frailty as a better predictor of morbidity and mortality for patients undergoing microsurgical resection of VS.

Regarding LOS, multiple studies have produced similar results. Average LOS after microsurgery for VS has been reported between 2 and 5.3 days,^2^, ^9^, ^17^, ^18^, ^19^, ^20^ with one study using >2 days as the cutoff for an increased LOS.^15^ Previous studies have listed increasing frailty, increased surgical time, and surgical complications as factors that increase length of stay.^9^, ^11^, ^18^, ^20^, ^21^, ^22^, ^23^ Other studies, in contrast to our own, have shown an association with female sex, increasing age, brainstem compression, tumor volume, non‐white race, and postoperative vertigo.^15^, ^17^, ^18^, ^20^ Additional factors with a reported association not examined here include low facility case volume,^24^, ^25^ coronary artery disease, and hypertension, though some of these comorbidities are included in the CCI or mFI‐11.^18^ BMI has not been reported as a factor increasing LOS.^26^ Our study found that frailty, surgical time, and surgical complications were significant predictors of LOS, consistent with previously published literature.^9^, ^11^, ^18^, ^20^, ^21^, ^22^ Other factors that were nonsignificant in the present study despite significance in prior studies may be due to limitations inherent to the cohort studied. Being able to predict postoperative length of stay may be useful for patient counseling regarding expected postoperative course and estimating financial impact, as LOS can be used as a proxy for total treatment cost.

There are fewer published data on factors associated with NHD in VS. Between 2002 and 2014, the NHD percentage after microsurgery for VS increased from 5.5% to 13.6%.^19^ A study by Ahmed et al in 2014 reported a 91.4% home discharge rate after resection of VS.^27^ High volume centers have lower rates of NHD^25^, ^27^, ^28^, ^29^; the present study is a single‐institution study, and therefore, did not address this question. Two recent studies have found an association between the VS‐5 index and nonroutine discharge (defined as a discharge location other than the patient's residence).^9^, ^30^ The VS‐5 index was proposed in 2022 and has limited research supporting its use. It has five criteria: age > 60, hydrocephalus, preoperative cranial nerve palsy, history of diabetes mellitus, and history of hypertension, thus involving items included on the 5‐item modified frailty index and mFI‐11 as well as the CCI. Other measures of low physical health, similar to frailty measurements, have been associated with NHD.^26^ Routine discharge (discharge to the patient's residence) has been found to be less likely for patients with medical comorbidities and those with Medicaid.^27^, ^31^ It is apparent from the results of this study and others that reasons for an NHD are multifactorial and may prove difficult to modify. While not all factors mentioned in the literature were evaluated, this study found only the placement of a VP shunt to be predictive of an NHD.

Factors associated with surgical complications have been thoroughly discussed in the literature, with some of the most common being hypertension, diabetes, tobacco use, obesity, and age.^26^, ^32^, ^33^, ^34^, ^35^, ^36^, ^37^, ^38^ Surgical approach has also been listed as a factor,^39^ while Raghavan et al showed that the duration of surgery was not associated with complications.^23^ Other factors mentioned in the literature include low‐volume institutions, comorbidities, extent of resection, and tumor size.^33^, ^40^, ^41^ Our study did not show associations after analysis between surgical complications and any of the factors examined, including frailty and CCI, with their corresponding comorbidities.

Findings from this study may influence factors such as resource allocation, patient counseling, and estimation of financial impact. To decrease LOS, mFI‐11 may be improved by delaying surgery (when able) until more than 6 months after a patient has had a major acute medical condition such as a myocardial infarction or pneumonia. Preoperative planning and efficient surgery can also decrease LOS.

### Strengths and Limitations

Strengths of this study include it being a single‐institution study with direct access to patient data and strong Pearson correlations for measured imaging data. In addition, the population of patients treated for large tumors is sizable, and the treatment team managing large VS at our institution is consistent and experienced

Limitations of this study include the retrospective nature of the study and the quality of data in the medical record pertaining to frailty scores. Additionally, there are limitations associated with the imaging modalities. Imaging was performed at multiple facilities with different MRI techniques in use. It is difficult to estimate the impact these differences may have had on the analyses central to this study, as imaging data were only one component. Another limitation is generalizability. Our cohort was primarily white and reflects the population served by the study institution. The results may be less applicable to institutions that serve more diverse populations. Lastly, there are additional variables that may influence patient outcomes that are not included in this study, such as pretreatment of dizziness with gentamicin and vestibular physical therapy.

## Conclusion

Frailty, as measured by an increased mFI‐11, surgical time, and surgical complications were independent predictors of increased LOS. VP shunt was predictive of NHD. There were no other independent predictors of increased LOS, NHD, or surgical complications. This evidence may impact patient education and counseling.

## Author Contributions

**Jason L. Steele**, Study design, data collection, analysis, manuscript preparation; **Nicole J. Ewer**, Study design, data collection, analysis, manuscript preparation; **Heather J. Smith**, Study design, data collection, analysis, manuscript preparation; **Samira Takkoush**, Data collection, analysis, manuscript preparation; **Melissa Shuhui Lee**, Data collection, analysis, manuscript preparation; **Richard H. Wiggins**, Study design, data collection, analysis, manuscript preparation; **Mana Espahbodi**, Study design, data collection, analysis, manuscript preparation; **Neil S. Patel**, Study design, data collection, analysis, manuscript preparation; **Richard K. Gurgel**, Study design, data collection, analysis, manuscript preparation.

## Disclosures

### Competing interests

Richard K. Gurgel, Advanced Bionics (grant/research support), Cochlear Americas (grant/research support), Med‐El (advisory committee/board member), and Neosensory (grant/research support). Neil S. Patel, Cochlear Corp (grant/research support), IotaMotion (speaker/honoraria), Viridian Therapeutics (consultant), and Zeiss (speaker/honoraria).

### Funding source

None.
